# FGF-2–Overexpressing Adipose-Derived Stem Cells as a Paracrine Platform for Angiogenesis-Driven Tissue Regeneration

**DOI:** 10.1007/s12195-025-00883-w

**Published:** 2025-12-29

**Authors:** Daisuke Seki, Michiyo Honda

**Affiliations:** Department of Applied Chemistry, School of Science and Technology, Meiji University, 1-1-1 Higashimita, Tama-ku, Kawasaki, Kanagawa 214-8571 Japan

**Keywords:** FGF-2, Adipose-derived mesenchymal stem cells, Angiogenesis, Osteogenesis, Paracrine signal

## Abstract

**Background:**

The survival and function of three-dimensional tissues critically depend on the establishment of a functional vascular network that ensures oxygen and nutrient supply and waste removal. Insufficient vascularization leads to hypoxia, metabolic stress, and cell death, making angiogenesis a fundamental requirement for successful tissue regeneration. This requirement is particularly evident in highly vascularized tissues such as bone, where vascular networks closely regulate tissue metabolism and repair.

**Methods:**

Human adipose-derived mesenchymal stem cells (ASCs) were genetically modified to overexpress fibroblast growth factor 2 (FGF-2), a key regulator of angiogenesis. The angiogenic potential of these cells and the paracrine effects of their conditioned medium were subsequently evaluated, together with their effects on osteogenic differentiation to assess functional specificity.

**Results:**

Overexpression of FGF-2 in ASCs enhanced endothelial cell migration and tube formation via paracrine mechanisms, in which elevated secretion of VEGFA and other angiogenic factors acted synergistically to promote angiogenesis. In contrast, osteogenic differentiation of ASCs was significantly inhibited by FGF-2 overexpression. Notably, *FGFR2* expression, the receptor for FGF-2, was selectively downregulated during osteogenic induction, suggesting that sustained FGF-2 signaling preferentially interferes with FGFR-mediated pathways associated with osteogenic maturation rather than with early proliferative responses.

**Conclusion:**

Our results demonstrate that FGF-2–overexpressing ASCs function not as osteogenic effector cells but as a potent paracrine platform for angiogenesis. Their conditioned medium, enriched with FGF-2 and synergistic angiogenic factors, supports vascular network formation and indirectly enhances the regenerative microenvironment, highlighting its potential as a cell-free strategy for tissue regeneration.

## Introduction

Without adequate vascularization, tissues are deprived of sufficient oxygen and unable to efficiently remove metabolic waste, ultimately leading to tissue necrosis. Vascularization remains a critical focus in regenerative medicine; however, the development of mature and functional vasculature continues to pose significant challenges, limiting progress in the field [1]. Regenerated tissues engineered using biomaterials are often fabricated at clinically relevant sizes (0.1–10 cm); however, the diffusion of oxygen and nutrients from existing vasculature is restricted to approximately 100–200 µm [2]. As a result, native blood vessels are unable to sustain cell viability at the core of engineered tissues. This limitation stems from the intrinsic constraints of in vitro tissue development, where the diffusion range of oxygen is insufficient to replicate the complexity of natural, in vivo tissue regeneration [3]. Therefore, novel strategies are needed to achieve stable and functional angiogenesis that closely mimics the maturation and architecture of native vasculature, thereby enhancing the survival and functionality of regenerated tissues.

Among the approaches investigated to overcome this vascularization barrier, mesenchymal stem cells (MSCs) have emerged as particularly attractive candidates. MSCs can be isolated from various tissues, including bone marrow, adipose tissue, and dental pulp. These multipotent stem cells are capable of differentiating into tissue-specific lineages, such as osteogenic, adipogenic, and chondrogenic cells [4]. However, increasing evidence suggests that the therapeutic benefits of MSCs in damaged tissues are primarily mediated not through their direct differentiation but through their paracrine signaling activity. These paracrine effects modulate the local microenvironment by promoting angiogenesis, cell proliferation, and tissue-specific differentiation. Regenerative therapies that leverage MSC-derived paracrine signaling show considerable promise; nonetheless, their therapeutic efficacy may be further improved by strategies aimed at enhancing the secretion of key paracrine factors [5].

One promising strategy to enhance the pro-angiogenic potential of MSCs is the incorporation of growth factors that regulate both vascular and skeletal tissue development. Among these, fibroblast growth factor-2 (FGF-2) has received particular attention due to its multifunctional roles in angiogenesis, wound healing, embryogenesis, and endocrine regulation [6]. FGF-2 exerts its effects by binding to fibroblast growth factor receptors (FGFRs) on MSCs, thereby activating multiple downstream signaling pathways, including MAPK/ERK, PI3K/Akt, and JAK/STAT pathways, which collectively promote cell proliferation, survival, and angiogenic potential [7–9]. Moreover, FGF-2 has been shown to be indispensable for maintaining the undifferentiated state of stem and progenitor cells in vitro [10, 11]. Owing to these diverse biological functions, FGF-2 has emerged as a promising therapeutic candidate in regenerative medicine and several clinical applications have already been attempted. Its effective concentrations, however, vary considerably depending on the disease context and route of administration. For instance, local application of up to 0.3% (≈3 mg/mL) has been shown to significantly enhance periodontal tissue regeneration [12], while much lower doses (10–100 µg) administered intracoronarily or locally in cardiovascular trials have promoted therapeutic angiogenesis [13]. These clinical findings underscore the broad therapeutic potential of FGF-2 and support its role in driving angiogenesis and tissue repair. Nevertheless, its therapeutic potential is limited by its inherent instability: FGF-2 is highly labile at physiological temperature (37 °C) [11], and its short in vivo half-life of approximately 12 hours [14] further constrains its effectiveness in sustained therapeutic contexts. Unlike the administration of a single growth factor, MSC–derived conditioned medium (CM) provides a multifunctional angiogenic microenvironment enriched with factors such as FGF-2 and VEGF, thereby supporting physiological angiogenesis from early endothelial activation through subsequent vessel maturation [5, 15]. Moreover, the controlled in vivo degradation of CM components enables temporally restricted angiogenic stimulation, ensuring adequate vascular support for bone regeneration while minimizing the risk of excessive or aberrant neovascularization. Notably, angiogenesis can be achieved at concentrations substantially lower than those required for single-agent therapies, likely owing to synergistic interactions among multiple angiogenic mediators.

In the present study, we generated *FGF-2*–overexpressing human adipose-derived MSCs (FGF-2_ASCs) via lentiviral transduction to enhance the pro-angiogenic properties of the MSC secretome. Rather than relying on direct cellular differentiation, we focused on evaluating the angiogenic activity mediated by paracrine signaling, together with the effects of sustained FGF-2 secretion on osteogenic differentiation. By enabling continuous and localized delivery of FGF-2 within a multifunctional secretome, this strategy addresses the short half-life of exogenously administered growth factors and provides a stable platform for angiogenesis-driven tissue regeneration based on MSC-derived CM.

## Materials and Methods

### Cell Culture

Commercially available human adipose-derived mesenchymal stem cells (ASC/TERT1, EVERCYTE, Vienna, Austria; Cat. No. CHT-001-0005) were cultured in Mesenchymal Stem Cell Growth Medium 2 (Takara Bio Inc., Shiga, Japan; Cat. No. C-28009) at 37 °C in a humidified atmosphere containing 5% CO_2_. For all experiments, ASC/TERT1cells were used between passages 3 and 7 after thawing from the original frozen stocks. Human umbilical vein endothelial cells (HUVEC; Takara Bio Inc., Cat. No. C-12203) were maintained in Endothelial Growth Medium 2 (Takara Bio Inc., Cat. No. C-22111) at 37 °C in a humidified atmosphere containing 5% CO₂. Only cells at passages ≤ 3 were used for all experiments.

### Virus-Mediated Gene Overexpression of *FGF2* in MSC

The viral vectors pLV[Exp] EGFP:T2A:Puro EF1A > hFGF-2, and pLV[Exp]-EGFP:T2A:Puro-EF1A > mCherry (control vector) were purchased from VectorBuilder (Chicago, IL, USA). To achieve lentiviral infection, ASCs were transduced with viral supernatant (MOI = 20) combined with Polybrene (10µg·mL^–1^, VectorBuilder) for 24 h, after which the culture medium was changed with fresh medium. After 48 h of transduction, 1 µg·mL^–1^ puromycin (Fujifilm Wako, Osaka, Japan; Cat. No. 160-23151) was added to select stable cell lines. The reporter lentiviral vector was used as a negative control (LV-Ct). These cells were characterized at both the mRNA and protein levels.

### Cell Proliferation Assay

Cell proliferation was assessed using the MTT assay. ASCs were seeded into 48-well plates at a density of 1 × 10^4^ cells per well in 300 µL of Mesenchymal Stem Cell Growth Medium 2 (Takara Bio Inc.). Cells were incubated at 37 °C in a humidified atmosphere containing 5% CO₂ for designated time points (1, 3, 5, 7 days). At each time point, 30 µL of MTT solution (5 mg/mL in phosphate-buffered saline (PBS), Sigma-Aldrich, Saint Louis, MO, USA; Cat. No. M2128) was added to each well, and the plates were incubated for 3 h at 37 °C. Following incubation, the medium was carefully removed, and 500 µL of dimethyl sulfoxide (DMSO; Fujifilm Wako, Cat. No. 043-07216) was added to each well to dissolve the formazan crystals. The absorbance was measured at 570 nm using a microplate reader (Multiskan^TM^ FC Microplate Photometer, Thermo Fisher Scientific, Waltham, MA, USA). Background absorbance at 620 nm was subtracted from the readings. Cell proliferation was calculated from the MTT assay using a standard curve. The measurements were performed in triplicate.

### Preparation of Conditioned Medium (CM)

The total cell density was 1 × 10^5^ cells/well in a 6-well plate and cultured in LV-infected ASCs for 24 h. After washing with PBS, the medium was replaced with serum-free DMEM (Gibco, Thermo Fisher Scientific, Cat. No. 11965-092) and the cells were cultured for 48 h. The supernatant was collected in 2.0-mL tubes and centrifuged (1,677 g for 5 min). The resulting material was filter-sterilized, collected in 2.0-mL tubes, and stored at − 80 °C. Hereafter, these samples are referred to as conditioned medium (CM).

### Cell Migration Assay (Wound Healing Assay)

The migratory capacity of the HUVECs treated with CM was evaluated using a scratch assay. Cells were seeded in 24-well plates at a density of 1 × 10^5^ cells per well and cultured in ECGM 2 (Takara Bio Inc.) until reaching 90–100% confluency. A sterile 200-μL pipette tip was used to create a straight scratch (wound) across the cell monolayer. The detached cells were removed by gentle washing twice with PBS, and the medium was replaced with CM. Images of the scratched area were captured immediately (0 h) and at designated time points (6 and 12 h) using an inverted phase-contrast microscope (CKX53, Olympus, Tokyo, Japan). The wound area was measured using the ImageJ software (Bethesda, MD, USA), and the percentage of wound closure was calculated as follows:

$${\mathrm{Wound}}\,\,{\mathrm{Closure}}\,\,\left( \% \right) = \left[ {\left( {{\mathrm{Initial}}\,\,{\mathrm{wound}}\,\,{\mathrm{area}} - {\mathrm{Wound}}\,\,{\mathrm{area}}\,\,{\mathrm{at}}\,\,{\mathrm{time}}\,\,{\mathrm{t}}} \right)/{\mathrm{Initial}}\,\,{\mathrm{wound}}\,\,{\mathrm{area}}} \right] \times {1}00$$

All experiments were performed in triplicate.

### Tube Formation Assay

Matrigel^®^ Basement Membrane Matrix (Corning Inc., Corning, NY, USA; Cat. No. 354234) was thawed on ice overnight and added (150 µL per well) to pre-chilled 48-well plates, followed by incubation at 37 °C for 30 min to allow gel polymerization. HUVECs were seeded onto the Matrigel-coated wells at a density of 5 × 10^4^ cells per well in CM collected from either FGF-2-overexpressing ASCs or control ASCs. ECGM-2 supplemented with endothelial growth factor was used as the positive control. Cells were incubated at 37 °C with 5% CO₂ for 14 h.

Tube formation was visualized under an inverted phase-contrast microscope (CKX53, Olympus), and images were captured in five random fields per well. Quantitative analysis was conducted using ImageJ software (NIH, Bethesda, MD, USA) with the Angiogenesis Analyzer plugin to assess the total tube length and number of branches.

### Quantitative Real-Time PCR (qRT-PCR)

The expression of marker genes, such as *FGF-2*, *VEGF*, *angiopoietin-2* (*ANGPT2*), *Tie2*, *alkaline phosphatase* (*ALP*), *osteocalcin* (*OC*), and *FGFR2*, was examined via qRT-PCR. The cells were seeded in each well of a 24-well plate at a total density of 1 × 10^5^ cells per well. The culture medium was refreshed every two days, and the culture was maintained for a maximum of 21 days. Total RNA was extracted using a FastGene RNA Basic Kit (Nippon Genetics Co., Ltd., Tokyo, Japan; Cat. No. FG-80250). Single-stranded complementary DNA was synthesized using the Transcriptor First-Strand cDNA Synthesis Kit (Thermo Fisher Scientific, Waltham, MA, USA) according to the manufacturer’s protocol. Primers used in the experiments are listed in Table 1. The quantitative-PCR analysis was conducted on a StepOne^®^ Real-Time PCR System (Thermo Fisher Scientific, Cat. No. 4371433) using SYBR^®^ Green Reagents (Takara Bio Inc., Cat. No. RR430A). The amount of mRNA was calculated as the amount relative to *GAPDH* and analyzed using the 2^-∆∆Ct^ method.

**Table 1:** Primers used in this study

Gene	Forward (5’ − 3’)	Reverse (5’ − 3’)
*FGF2*	GAGCGACCCTCACATCAA	CGTTTCAGTGCCACATACC
*VEGFA*	CGCAGCTACTGCCATCCAAT	GTGAGGTTTGATCCGCATAATCT
*ALP*	GCTGTAAGGACATCGCCTACCA	CCTGGCTTTCTCGTCACTCTCA
*ANGPT2*	GAACACTCCCTCTCGACAAACA	GATGATGTGCTTGTCTTCCATAGC
*Tie2*	CCATCACTCAGTATCAGCTCAAGGG	GCTTGACCCTATGTTGTTCTCTGC
*OC*	CCTCACACTCCTCGCCCTATT	CCCTCCTGCTTGGACAAA
*FGFR2*	GCACTGGAGCCTCATTATGGAA	ATCGCTCCACAACATCCAGGT
*GAPDH*	GTCTCCTCTGACTTCAACAGCG	ACCACCCTGTTGCTGTAGCCAA

### ELISA (Enzyme-Linked Immunosorbent Assay)

The culture supernatant was collected to determine the concentration of FGF-2 and VEGF in the culture supernatant under each culture condition. Cells were seeded at a density of 1 × 10^5^ cells per well in a 6-well plate and cultured in LV-infected ASC for 24 h. After washing with PBS, the medium was replaced with serum-free DMEM and the cells were cultured for 2 days. The supernatant was collected in 2.0-mL tubes and centrifuged (1,677 g for 5 min). The samples were filter-sterilized, collected in 2.0-mL tubes, and stored at − 80 °C. The concentrations of FGF-2 and VEGF were quantified using the Quantikine® Human FGF Basic Immunoassay (R&D Systems, Inc., Minneapolis, MN, USA; Cat. No. DFB50) and the Human VEGF ELISA Kit (Proteintech Group, Inc., Rosemont, IL, USA; Cat. No. KE00085), respectively, according to the manufacturers’ instructions.

### Osteoblastic Differentiation

LV-infected ASC were seeded at 1 × 10^5^ cells per well in 24-well plates with growth medium (GM) and precultured for 24 h. The medium was then replaced with differentiation induction medium (ODM; DMEM supplemented with Osteoblast Inducer Reagent; Takara Bio Inc., Cat. No. MK430), according to the manufacturer’s protocol. The culture medium was refreshed every 3 days, and the culture was maintained for a maximum of 21 days.

#### Picrosirius Red Staining

LV-infected ASCs were seeded at 1 × 10^5^ cells per well in 24-well plates with growth medium and precultured for 24 h. The medium was replaced with fresh DM. The culture medium was replaced every 3 days. After 7 and 14 days of culture, Sirius Red staining was performed using Picrosirius Red Stain Kit (ScyTek Laboratories, Logan, UT, USA; Cat. No. PSR-1) at 25 °C. Briefly, cells were fixed with 4% paraformaldehyde in PBS for 15 min followed by washing with ultrapure water and air-drying for 15 min. A total of 150 µL of phosphomolybdic acid was added to the samples and incubated for 2 min. After three washes with 200 µL of ultrapure water, 150 µL of Picro-Sirius Red F3BA solution was added and the samples were incubated for 1 h. After washing with ultrapure water, cells were treated with 0.1 M hydrochloric acid for 15 min. The samples were examined under a microscope after air drying for 15 min. Samples were treated with 0.01 M sodium hydroxide solution for 30 min for extraction of collagen. The absorbance of the extracted solution at 550 nm was measured with a spectrophotometer, with 0.01 M sodium hydroxide solution as the control.

#### Alizarin Red S Staining

LV-infected ASCs were seeded at 1 × 10^5^ cells per well in 24-well plates with GM and precultured for 24 h. Subsequently, the medium was replaced with DM or GM. The culture medium was replaced every 3 days. After 24 d of culture, the cells were washed twice with PBS and fixed with 4% paraformaldehyde in PBS for 15 min at room temperature. The cells were washed twice with dH_2_O and stained with 0.1% alizarin red S solution (pH 4.1; Fujifilm Wako, Cat. No. 011-01192) for 1 h at room temperature in the dark. The cells were washed four times with dH_2_O and examined under a fluorescence microscope (BZ X-710, Keyence, Osaka, Japan). Cetylpyridinium chloride (10 %, w/v; Fujifilm Wako, Cat. No. 190177) was added to a 24-well plate. Optical density was measured at 562 nm.

#### Statistical Analysis

Data were statistically analyzed to determine the mean and standard deviation (S.D.). Significant differences were determined using the GraphPad Prism software (version 10; GraphPad, San Diego, CA, USA). Student’s t-test was used to determine the statistical differences between two groups. Two-way analysis of variance (ANOVA) followed by Bonferroni multiple comparison post-hoc tests were used to compare the levels of different experimental groups. A probability value of *p* < 0.05 was considered statistically significant.

## Results

### Characterization of ASCs Overexpressing FGF2 (FGF-2_ASCs)

To generate stably expressing cell lines, ASCs were transduced with a lentiviral vector encoding either FGF-2 (LV-FGF-2) or a control reporter gene (LV-Ct), followed by puromycin selection. GFP expression was monitored over time to confirm successful transduction.

**Fig. 1:**
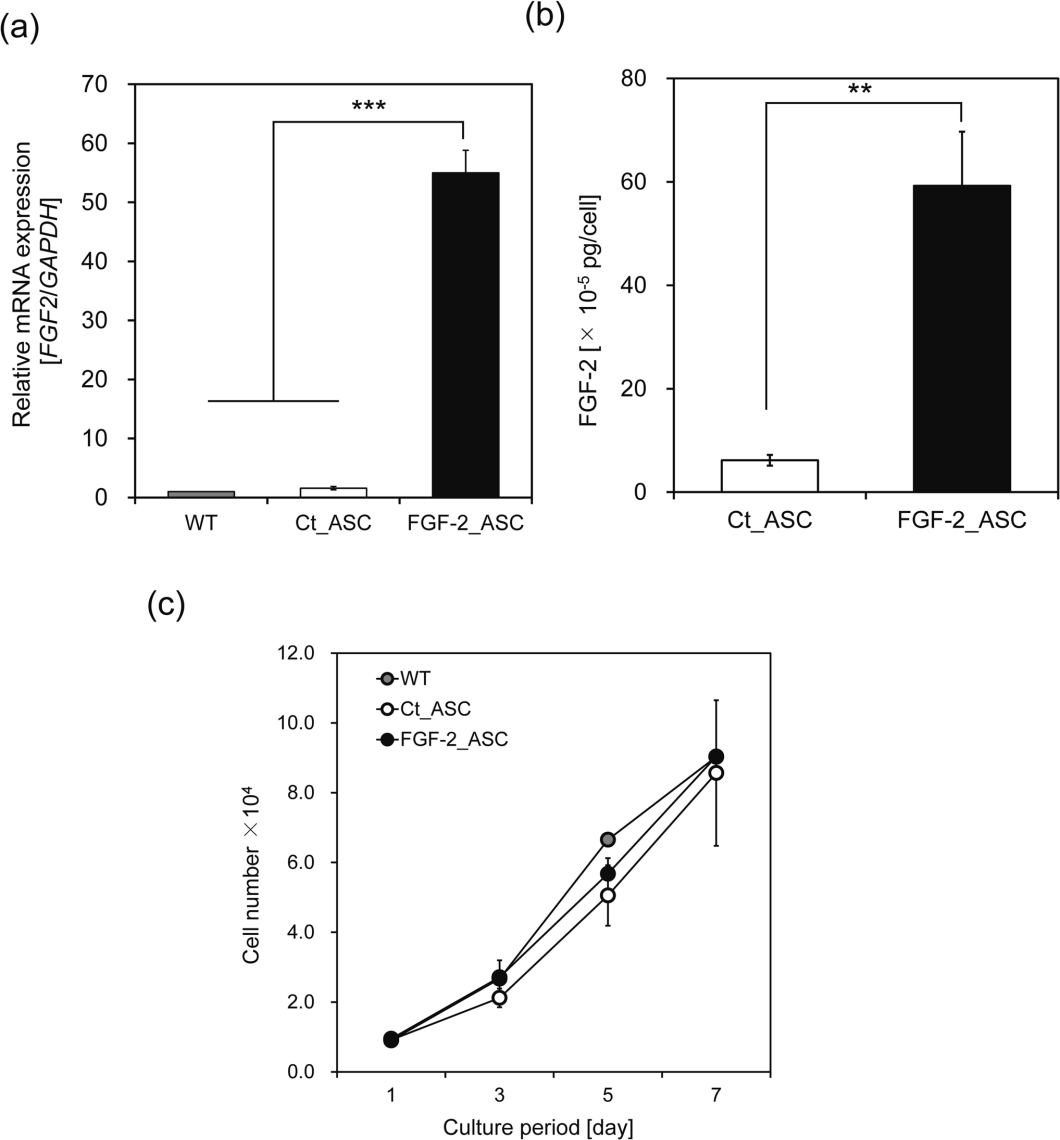
Characterization of FGF-2-overexpressing ASCs. FGF-2 expression and secretion were evaluated following lentiviral vector transduction, and the effect of FGF-2 overexpression on ASC proliferation was examined. **a** FGF-2 mRNA levels were quantified by qRT-PCR after 48 h of culture. Relative expression was calculated using the 2^-∆∆Ct^ method, normalized to GAPDH, and compared to non-transduced ASCs. **b** FGF-2 protein secretion was assessed by culturing cells in serum-free DMEM for 48 h, followed by ELISA of conditioned medium (CM). Background signals were corrected using CM from non-transduced cells. **c** Cell proliferation was measured by MTT assay in control and FGF-2-overexpressing ASCs cultured for 1, 3, 5, and 7 days. Formazan was solubilized in DMSO, and absorbance was recorded at 570 nm with background correction at 620 nm. n = 3; error bars represent ± S.D.; **p < 0.01, ***p < 0.001

Quantitative real-time PCR (qRT-PCR) revealed that *FGF-2* expression in FGF-2_ASCs was approximately 55-fold higher than in control cells (Ct_ASCs) (Fig. 1a). Consistent with these findings, ELISA results showed that the concentration of FGF-2 secreted by FGF-2_ASCs ((59.24 ± 10.47) × 10^-5^ pg·cell⁻^1^) was approximately 10 times higher than that secreted by Ct_ASCs ((6.17 ± 1.03) × 10^-5^ pg·cell⁻^1^) (Fig. 1b). These results confirm that lentiviral transduction with LV-FGF-2 effectively enhanced both the expression and secretion of FGF-2 in ASCs.

To assess the effect of FGF-2 on cell proliferation, an MTT assay was conducted (Fig. 1c). No significant differences in ASC proliferation were observed among the groups after 7 days (*p* > 0.05). In this study, ASCs were also transduced with a retroviral vector carrying the human telomerase reverse transcriptase (hTERT) gene to achieve cellular immortalization. Due to the absence of growth retardation or replicative senescence in these immortalized ASCs, *FGF-2* overexpression did not appear to affect cell proliferation. Since WT and Ct_ASCs showed no differences in FGF-2 expression or proliferative capacity, Ct_ASCs were used as the control for FGF-2_ASCs in subsequent experiments.

### Enhancement of Angiogenic Properties by Overexpression of FGF-2

**Fig. 2:**
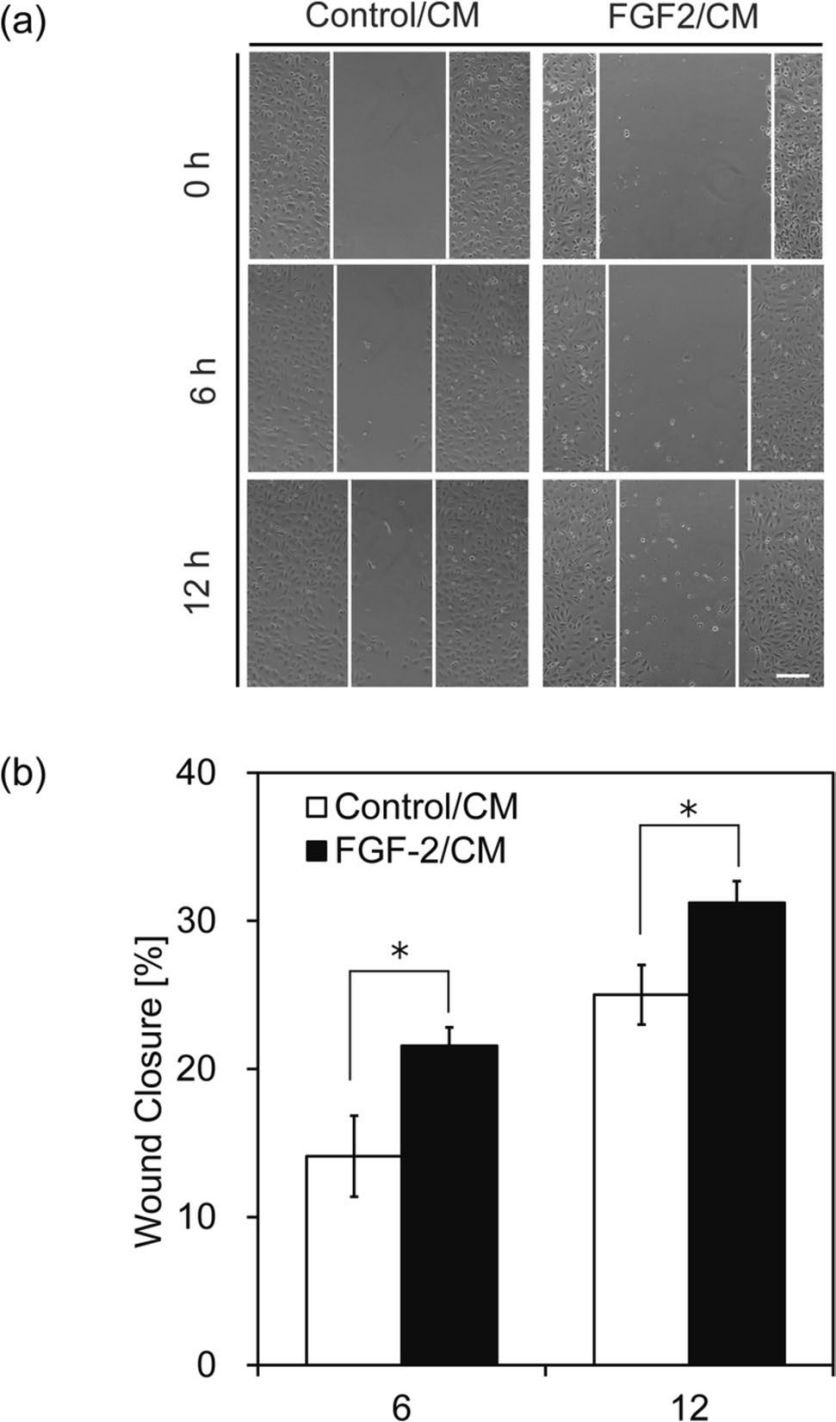
Enhanced HUVEC migration induced by CM from FGF-2-overexpressing ASCs. HUVEC migration was evaluated using a scratch assay with conditioned medium (CM) from control or FGF-2-overexpressing ASCs. **a** Representative images at 0, 6, and 12 h post-scratch. **b** Wound closure was quantified by measuring wound width at five randomly selected points and calculating the repair rate relative to the 0 h width. Scale bars = 200 µm; *n* = 3; error bars represent ± S.D.; *p < 0.05

**Fig. 3:**
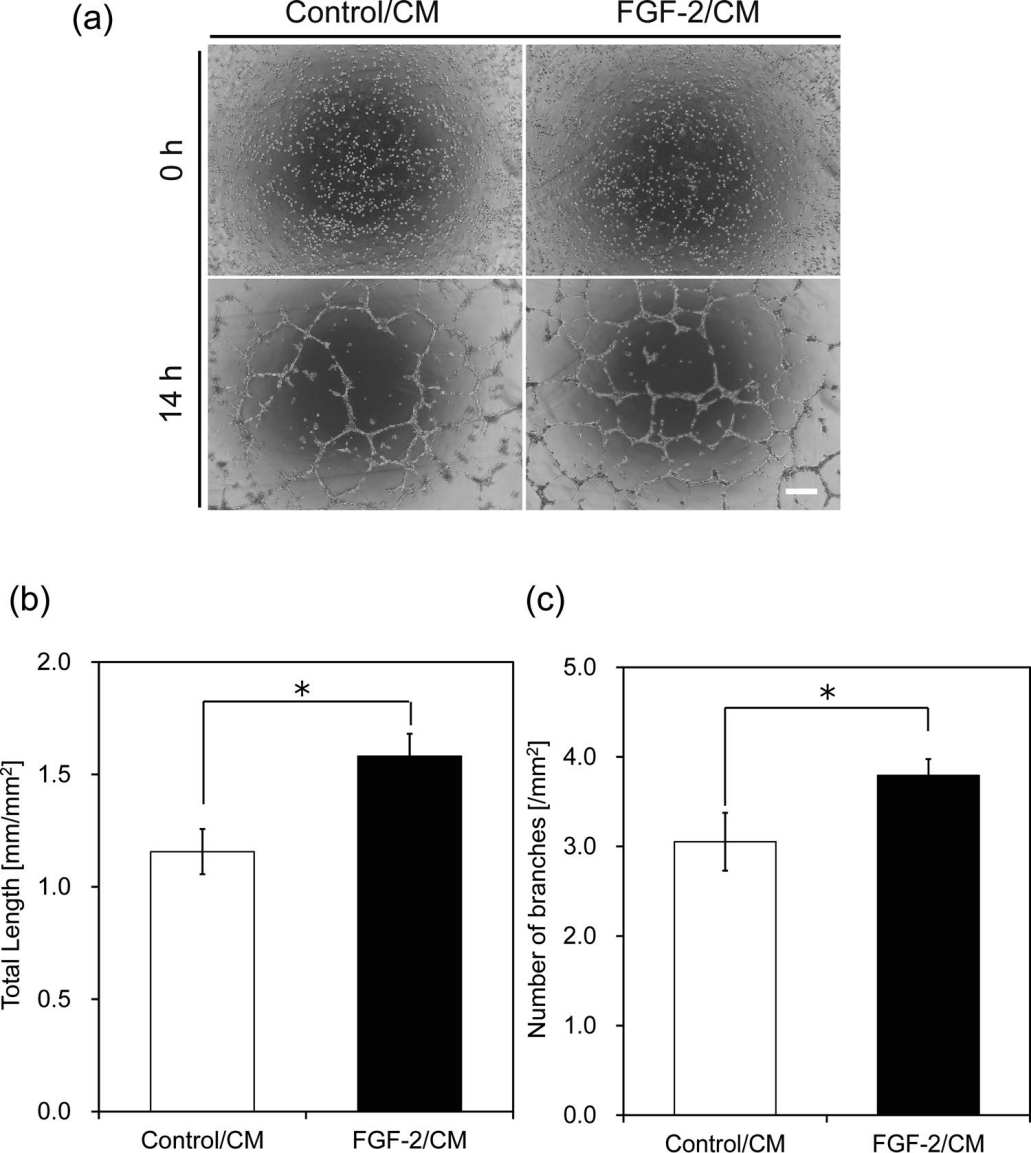
Promotion of tube formation by CM from FGF-2-overexpressing ASCs. Tube formation by HUVECs on Matrigel was assessed after treatment with CM from control or FGF-2-overexpressing ASCs. **a** Representative images at 0 and 14 h. Quantification was performed at 14 h using ImageJ on five randomly selected fields per sample. **b** Total tube length and **c** number of branch points were measured per image area. Scale bars = 200 µm; *n* = 3; error bars represent ± S.D.; *p < 0.05

**Fig. 4:**
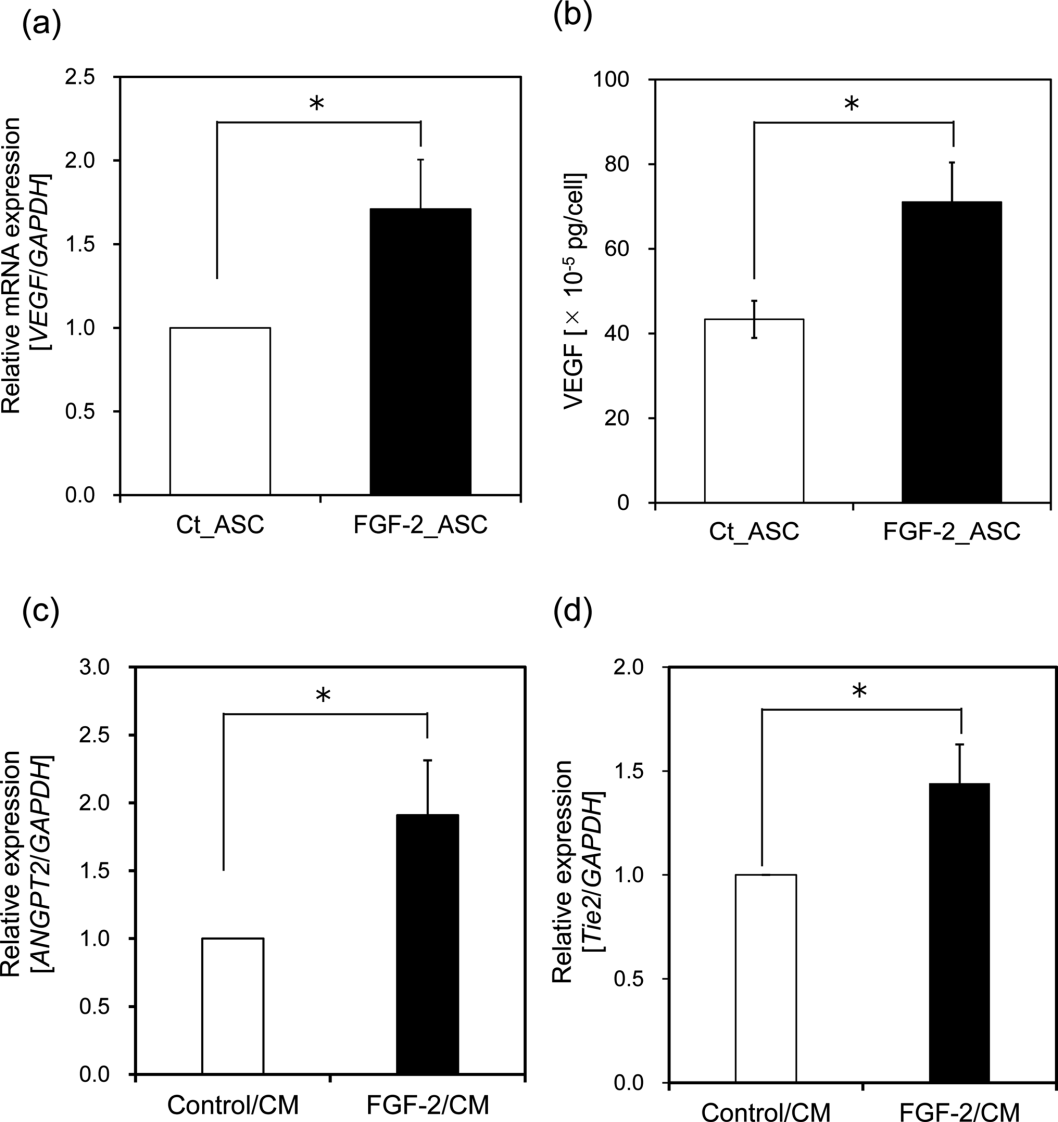
FGF-2 overexpression upregulates VEGFA expression in ASCs and alters gene expression in HUVECs cultured with ASC-conditioned medium. **A** VEGFA mRNA and protein levels in ASCs cultured for 48 h in serum-free DMEM. Relative *VEGFA* expression was measured by qRT-PCR and normalized to *GAPDH*. VEGFA secretion was quantified by ELISA of conditioned medium (CM), with background correction using serum-free DMEM. **B** Gene expression in HUVECs after 48 h of culture with CM from control or FGF-2-overexpressing ASCs. *ANGPT2* and *Tie2* mRNA levels were quantified by qRT-PCR, normalized to *GAPDH*, and calculated using the 2^-∆∆Ct^ method. *n* = 3; error bars represent ± S.D.; *p < 0.05

Exogenous FGF-2 treatment has demonstrated significant potential to stimulate angiogenesis and the formation of new blood vessels. To investigate this effect, we prepared conditioned media (CM) from Ct_ASCs and FGF-2_ASCs, and assessed their angiogenic potential in human umbilical vein endothelial cells (HUVECs). Specifically, we evaluated HUVEC proliferation in the respective CM (Ct_ASCs CM: Control/CM; FGF-2_ASCs CM: FGF-2/CM). A marked decrease in cell number was observed in the Control/CM group, while no significant change was noted in the FGF-2/CM group. These findings suggest that neither CM promoted HUVEC proliferation. As shown in Figure S1a, the concentration of FGF-2 secreted by FGF-2_ASCs was 82.6 pg·mL⁻^1^, a level that appears insufficient to stimulate proliferation in HUVECs. However, FGF-2/CM was able to sustain cell viability for up to three days, unlike Control/CM, which was associated with decreased cell survival. These results suggest that while the FGF-2 concentration in FGF-2/CM does not reach the threshold necessary for inducing proliferation, it is sufficient to support HUVEC survival under serum-reduced conditions. To evaluate the migratory capacity of HUVECs, a wound healing assay was conducted. As shown in Fig. 2, the percentage of wound closure was significantly higher in the FGF-2/CM group at all observed time points, indicating that FGF-2/CM enhances HUVEC motility. In addition to migration, tube formation is a key functional marker of angiogenesis. To compare the tube-forming potential of the CM, a Matrigel-based tube formation assay was performed. HUVECs were cultured on Matrigel-coated plates in the presence of either Control/CM or FGF-2/CM for 14 h (Fig. 3a). Micrographs were acquired post-incubation, and the resulting capillary-like networks were quantitatively analyzed using ImageJ software. HUVECs exposed to FGF-2/CM formed well-developed and interconnected networks of capillary-like structures, in contrast to those cultured in Control/CM (Fig. 3b, c). Previous studies have reported that FGF-2 concentrations exceeding 20 ng·mL⁻^1^ are effective in promoting tube formation in HUVECs cultured on Matrigel [16]. Although the concentration of FGF-2 in FGF-2/CM was substantially lower (82.6 ± 13.1 pg·mL⁻^1^), these results suggest that other paracrine factors secreted by FGF-2_ASCs may synergize with FGF-2 to support angiogenic activity. In the present study, the FGF-2 concentrations in CM (82.6 pg⸱mL^-1^) may not reach the levels necessary to promote angiogenesis in HUVECs. These results suggest that the overexpression of *FGF-2* in ASCs may influence the gene expression levels of other angiogenesis-related factors. Therefore, to investigate the factors responsible for the influence of ASCs on angiogenesis, we focused on VEGFA, another important angiogenesis-related factor, and performed a qRT-PCR analysis. The expression of *VEGFA* in FGF-2_ASC was 1.7 times higher than that in Ct_ASC (Fig. 4a). In addition, ELISA showed that the concentration of VEGFA secreted from FGF-2_ASCs was approximately 1.5 times higher than the corresponding result with Ct_ASCs (Fig. 4b). The pro-angiogenic effect of VEGFA on HUVECs has been observed at concentrations ranging from 10 to 50 ng⸱mL^-1^ [17]. Although VEGFA alone is insufficient to induce robust angiogenesis (Fig. S1b), its synergistic interaction with FGF-2 has been shown to significantly enhance angiogenic responses in HUVECs [18, 19].

To investigate the impact of CM on the expression of angiogenesis-related genes, HUVECs were cultured in Control/CM or FGF-2/CM for 48 h, followed by qRT-PCR analysis. We focused on *angiopoietin-2* (*ANGPT2*) and its receptor *Tie2*, both of which play key roles in regulating endogenous angiogenesis [20]. As shown in Fig. 4c and d, the expression levels of *ANGPT2* and *Tie2* were elevated in HUVECs cultured in FGF-2/CM compared to those in Control/CM. This suggests that FGF-2 and VEGFA present in the CM stimulate HUVECs to upregulate *ANGPT2* expression. In turn, increased *ANGPT2* may further activate *Tie2* signaling in an autocrine or paracrine manner, thereby contributing positively to angiogenic processes.

### Inhibition of ASCs Osteogenesis by FGF-2 Overexpression

**Fig. 5.**
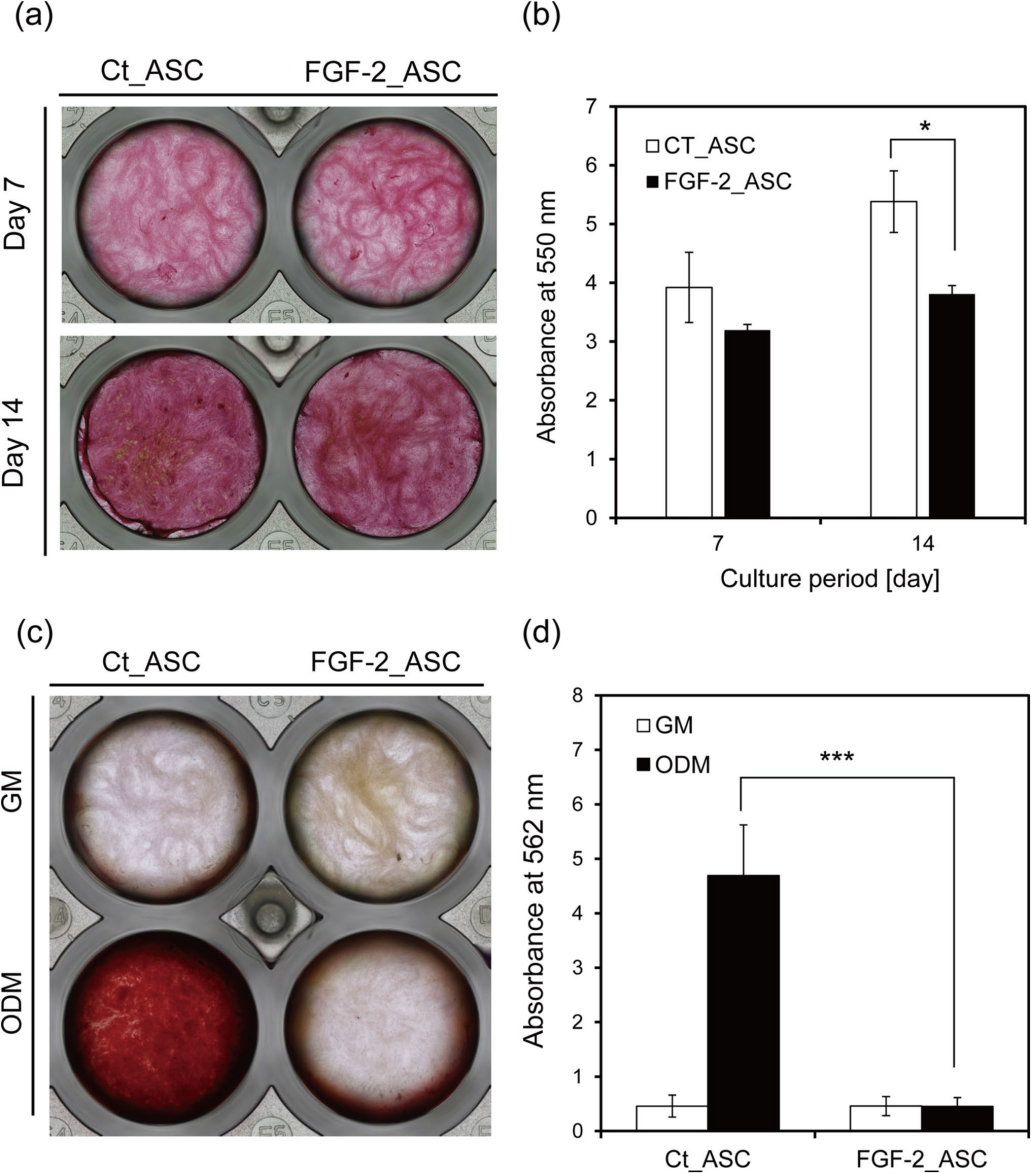
Suppression of collagen production and osteogenic differentiation by FGF-2 overexpression in ASCs. **a** Representative images of Picrosirius Red-stained ASCs after 7 or 14 days of culture in osteogenic differentiation medium (ODM). **b** Quantification of collagen content by dye extraction with 0.1 M sodium hydroxide and absorbance measurement at 550 nm. **c** Representative images of Alizarin Red S-stained ASCs after 24 days of culture in growth medium (GM) or ODM. **d** Quantification of calcium deposition by dye extraction with 10% CPC solution and absorbance measurement at 562 nm. n = 3; error bars represent ± S.D.; *p < 0.05, ***p < 0.001

Previous studies have revealed that FGF-2 both promotes and inhibits bone differentiation [21, 22]. To examine the impact of *FGF-2* overexpression on osteogenesis, we quantified collagen production—a key marker of osteogenic activity—using Picrosirius Red staining (Fig. 5a). Collagen deposition increased over time in both FGF-2_ASCs and Ct_ASCs during the differentiation period. However, by day 14, collagen production was significantly lower in FGF-2_ASCs compared to Ct_ASCs, suggesting that *FGF-2* overexpression may inhibit late-stage osteogenic differentiation (Fig. 5b).

Alizarin Red S staining was performed on day 24 to examine the effect of FGF-2 on calcium deposition. As shown in Fig. 5c and d, Alizarin Red-stained calcium deposits were observed in Ct_ ASCs-induced osteogenic differentiation; however, no calcium deposits were observed in the FGF-2_ASCs group. These results indicate that overexpression of *FGF-2* in ASCs inhibits their osteogenic differentiation.

**Fig. 6:**
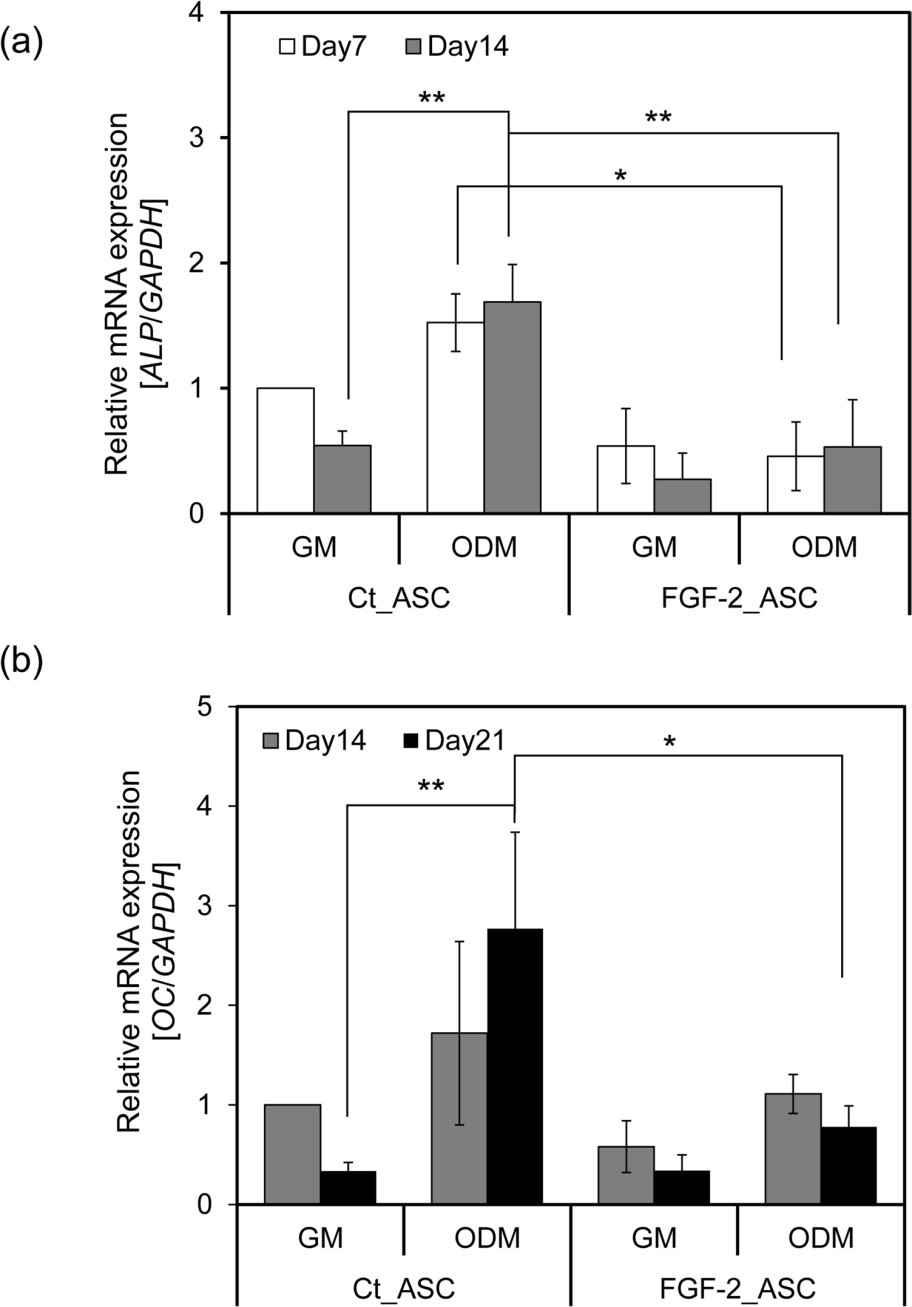
Effects of FGF-2 overexpression on osteogenic marker expression. *ALP* and *Osteocalcin* (*OC*) expression were analyzed by qRT-PCR in ASCs cultured in GM or ODM. **a** *ALP* expression was evaluated after 7 or 14 days and **b** *OC* after 14 or 21 days. Expression levels were normalized to *GAPDH* and calculated relative to day 7 (*ALP*) or day 14 (*OC*) control-GM samples. *n* = 3; error bars represent ± S.D.; *p < 0.05, **p < 0.01

qRT-PCR was conducted to assess the expression levels of osteogenic marker genes. *Alkaline phosphatase* (*ALP*), an early marker of osteogenic differentiation, showed increased expression in Ct_ASCs following 14 days of osteoinductive culture. In contrast, FGF-2_ASCs exhibited no significant change in *ALP* expression under either growth medium (GM) or osteogenic differentiation medium (ODM) conditions (Fig. 6a). Moreover, under osteoinductive conditions, *ALP* expression in FGF-2_ASCs was significantly lower than in Ct_ASCs, indicating that FGF-2 overexpression suppresses the early phase of osteogenic differentiation. To further evaluate the impact of FGF-2 on osteogenesis, the expression of *osteocalcin* (*OC*)—a late-stage marker of osteogenic maturation—was analyzed (Fig. 6b). In Ct_ASCs, *OC* expression markedly increased by day 21 under osteoinductive conditions, while in FGF-2_ASCs, *OC* expression remained significantly lower. These results suggest that the suppression of early osteogenic markers, such as *ALP*, in FGF-2_ASCs may lead to a downstream reduction in late-stage differentiation markers like *OC*. Collectively, these findings indicate that *FGF-2* overexpression inhibits osteogenic differentiation at both early and late stages.

### FGF-2 Receptor (FGFR2) is Related to Osteogenesis

**Fig. 7:**
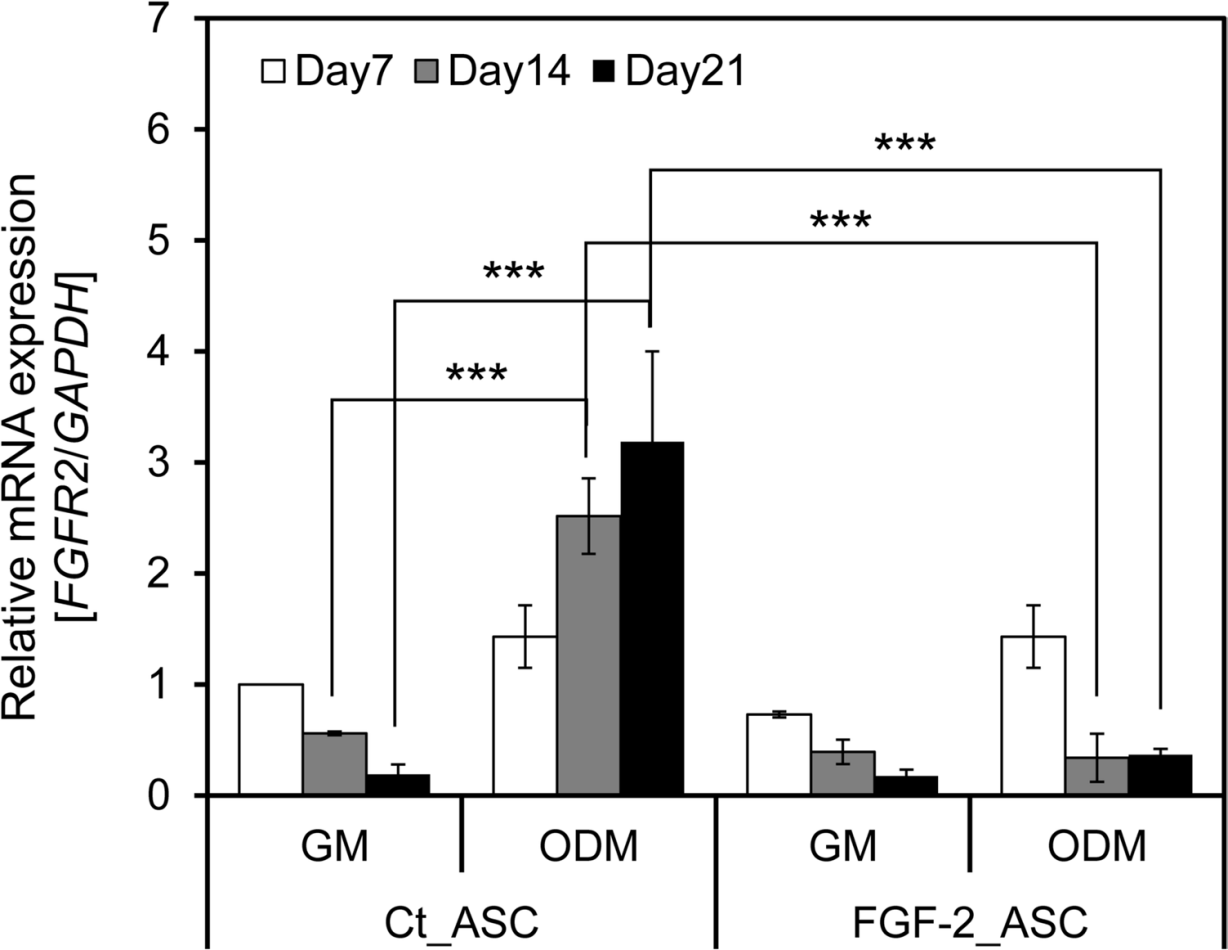
*FGFR2* expression in FGF-2-overexpressing ASCs during osteogenic differentiation. *FGFR2* expression was quantified by qRT-PCR in ASCs cultured in GM or ODM for 7, 14, or 21 days. Expression levels were normalized to *GAPDH* and compared to day 7 control-GM conditions. *n* = 3; error bars represent ± S.D.; ***p < 0.001

To elucidate the mechanism underlying the suppression of osteogenic differentiation by FGF-2 overexpression, we investigated the expression of *fibroblast growth factor receptor 2* (*FGFR2*). FGFR2 is known to play a pivotal role in osteogenesis by regulating signaling pathways involved in bone formation and differentiation. Previous studies have demonstrated that FGFR2 positively regulates osteogenic markers, including ALP, and is positively correlated with osteogenic activity [23]. Additionally, it has been reported that exogenous FGF-2 can downregulate *FGFR2* expression [24]. In the present study, we quantified *FGFR2* mRNA levels using qRT-PCR (Fig. 7). In Ct_ASCs, *FGFR2* expression was significantly upregulated after 14 days of osteoinductive culture, consistent with the observed increase in *ALP* expression. In contrast, FGF-2_ASCs showed a significant reduction in *FGFR2* expression under the same conditions, despite the presence of osteogenic stimuli. These findings suggest that *FGF-2* overexpression suppresses osteogenic differentiation by downregulating *FGFR2* expression, thereby interfering with receptor-mediated signaling essential for bone formation. Our results align with previous reports emphasizing the critical role of FGFR2 in regulating osteogenic differentiation [23].

## Discussion

In this study, we generated FGF-2-overexpressing ASCs (FGF-2_ASCs) and evaluated their angiogenic and osteogenic potential. *FGF-2* expression in FGF-2_ASCs was approximately 55-fold higher than in control cells, and the concentration of secreted FGF-2 reached approximately 80 pg·mL⁻^1^. While FGF-2 is generally known to enhance cell proliferation, no significant change in proliferation was observed in our experiments. This lack of effect is likely attributable to the use of immortalized ASCs, which exhibit sustained proliferative capacity and are less responsive to external mitogenic stimuli. FGF-2 is known to act as a potent mitogen for primary MSCs, particularly during early passages, where it transiently stimulates cell cycle progression via activation of the MAPK and PI3K/Akt signaling pathways [25]. However, this mitogenic effect is largely dependent on exogenous supplementation and diminishes upon withdrawal of FGF-2 or prolonged culture. In contrast, telomerase reverse transcriptase (TERT) overexpression intrinsically sustains proliferative capacity by stabilizing telomere length and maintaining cell cycle gene activation [26]. Thus, in TERT-expressing MSCs, the proliferative potential is maintained independently of exogenous mitogenic stimuli. Accordingly, the contribution of FGF-2 to cell proliferation becomes relatively minor compared with the intrinsic growth-supporting effects of telomerase activation.

To assess the angiogenic potential of the CM, HUVECs were cultured in either Control/CM or FGF-2/CM, and evaluated for key angiogenic behaviors, including proliferation, migration, and tube formation. In this study, no significant increase in HUVEC proliferation was observed, likely due to the relatively low concentration of FGF-2 (approximately 80 pg·mL⁻^1^) in the FGF-2/CM. Previous studies have demonstrated that FGF-2 concentrations of at least 25 ng·mL⁻^1^ are required to stimulate significant endothelial cell proliferation. Therefore, the concentration of FGF-2 in the CM used here was likely below the threshold necessary to elicit a proliferative response [27]. In addition to FGF-2, other growth factors such as epidermal growth factor (EGF) and insulin-like growth factor (IGF), which are commonly present in endothelial cell growth media, are known to promote cell proliferation. However, in the present study, it is likely that the concentrations of these factors in the CM did not reach the levels required to stimulate a proliferative response. In contrast, treatment with FGF-2/CM significantly enhanced HUVEC migration. Notably, previous studies have shown that low concentrations of FGF-2 can influence cell motility; for example, stimulation with vaccarin—a compound known to improve blood circulation—has been reported to increase cell migration by elevating FGF-2 levels to approximately 12 pg·mL⁻^1^. This suggests that even sub-threshold concentrations of FGF-2, while insufficient to induce proliferation, may still be effective in promoting endothelial cell migration [28]. These results are in line with previous evidence that sub-nanomolar concentrations of FGF-2 preferentially activate migratory and cytoskeletal remodeling pathways without inducing proliferation. For instance, low-dose FGF-2 selectively triggers focal adhesion kinase (FAK) and Rac1 activation, enhancing endothelial motility while bypassing ERK-mediated cell cycle entry [29]. This differential sensitivity suggests that the biological outcome of FGF-2 stimulation depends on both its concentration and the cellular context. Matrigel tube formation assays demonstrated that a denser capillary-like network was formed in the FGF-2/CM group, likely due to the secretion of FGF-2 and other angiogenic factors. It is well established that the secretory profile of MSCs can be modulated by external stimuli [30], which may, in turn, influence the expression of other angiogenesis-related genes. Previous studies have reported that exogenous FGF-2 enhances the autocrine and paracrine secretion of VEGFA through activation of the PI3K signaling pathway [31, 32]. In our study, FGF-2_ASCs were activated via similar signaling mechanisms. Although the concentrations of secreted VEGFA and FGF-2 alone were insufficient to significantly enhance angiogenesis, the combination of FGF-2 and ASCs (FGF-2 + ASC) promoted the formation of capillary-like structures. These findings suggest that the synergistic interaction between FGF-2 and VEGFA contributes to enhanced angiogenic activity. The synergistic effect between FGF-2 and VEGFA observed here has also been widely documented in angiogenic research. FGF-2 not only enhances VEGFA expression through PI3K/Akt and MAPK/ERK signaling but also increases VEGF receptor-2 (VEGFR2/KDR) availability on endothelial cells [33–35]. Conversely, VEGFA can augment FGF receptor signaling by promoting receptor dimerization and downstream phosphorylation events, forming a reciprocal amplification loop. Such cross-activation facilitates endothelial sprouting, lumen formation, and stabilization of neovessels, leading to more mature vascular networks than either factor alone. Therefore, the enhanced tube formation in our FGF-2/CM group likely reflects this integrated signaling, in which FGF-2 primes both ASCs and endothelial cells for VEGFA-mediated angiogenesis. To further investigate how CM influences the expression of angiogenesis-related genes in HUVECs, we focused on ANGPT2 and its receptor Tie2. ANGPT2, which increases vascular permeability, is typically expressed in tissues undergoing vascular remodeling. Its expression is also induced by cytokines such as FGF-2, VEGFA, and PDGFB [20, 36]. Activation of HUVECs by FGF-2 and VEGFA in FGF-2/CM resulted in increased expression of *ANGPT2* and *Tie2*. Exogenous ANGPT2 activates the receptor Tie2 on endothelial cells to a lesser extent than endogenous ANGPT2 on endothelial cells [20]. Endogenous ANGPT2 positively regulates angiogenesis by activating Tie2 signaling in HUVECs. Consistent with these findings, our data suggest that MSC-derived secreted factors, most likely including FGF-2, act in a paracrine manner to remodel the ANGPT/Tie2 signaling axis in endothelial cells. Previous studies have demonstrated that VEGF and FGF-2 are potent inducers of ANGPT2 expression in endothelial cells [37, 38], and that FGF-2 can modulate Tie2 expression in microvascular endothelial cells [39]. Our results extend these observations by showing that the ASC secretome is sufficient to promote the simultaneous induction of ANGPT2 and Tie2 in HUVECs. Importantly, the concurrent upregulation of both ligand and receptor may not simply reflect an antagonistic interaction but rather a context-dependent reorganization of the signaling network, enabling endothelial cells to balance destabilizing and stabilizing cues. Such dual induction may provide the endothelial plasticity necessary for effective angiogenic remodeling. Collectively, these data support the concept that ASCs exert pro-angiogenic and vessel-stabilizing effects not only through direct cell–cell interactions but also via paracrine modulation of endothelial signaling pathways.

These data demonstrated that TERT-expressing MSCs engineered to overexpress FGF-2 provide a robust and stable platform for the continuous production of pro-angiogenic CM. By preventing senescence-associated alterations in the MSC secretome, this strategy preserves angiogenic activity, while FGF-2 overexpression enables potent induction of early-stage angiogenesis at markedly lower effective concentrations than recombinant FGF-2.

To clarify the role of FGF-2 in bone metabolism, we investigated its effects on osteoblastic differentiation. Previous studies have reported conflicting findings regarding the impact of FGF-2 on bone formation [21]. While some studies suggest that FGF-2 promotes bone formation and regeneration [40], others report that it inhibits bone formation and suppresses ossification [41]. Bone formation at the site of injury typically occurs in two distinct phases: proliferation and calcification. These phases are regulated by dynamic changes in gene expression. Growth factors such as FGF-2 are known to initiate the proliferative phase, during which cell division and DNA synthesis are actively promoted. This is followed by the calcification phase, in which cell proliferation slows, and mineralization processes are initiated [42]. Long-term overexpression of FGF-2 may prolong the proliferative phase and inhibit osteogenic differentiation. In this context, we focused on FGFR2, which has been implicated in the suppression of osteogenesis. Notably, *FGFR2* expression showed a positive correlation with *ALP* expression, a marker of early osteogenic differentiation. In our study, *FGFR2* expression was upregulated in control ASCs (Ct_ASCs) cultured in ODM, whereas overexpression of FGF-2 (FGF-2_ASCs) led to a decrease in *FGFR2* expression (Fig. 7). These findings align with previous reports demonstrating that treatment with exogenous FGF-2 downregulates *FGFR2* expression [24]. Furthermore, knockdown of *FGFR2* has been shown to inhibit activation of the ERK signaling pathway and to suppress osteogenic differentiation [43], suggesting that FGFR2 is a key regulator in this process. Sustained autocrine FGF-2 signaling is thought to induce negative feedback via ERK-dependent transcriptional repressors or receptor internalization pathways. Continuous FGF-2 exposure has been reported to trigger receptor desensitization and lysosomal degradation of FGFR2 [44]. Such feedback regulation may protect cells from excessive mitogenic signaling but could also inadvertently impair osteogenic differentiation by attenuating downstream ERK and RUNX2 activation.

In this study, the transient suppression of *FGFR2* expression observed under FGF-2 overexpression does not represent a permanent alteration of MSC phenotype. In the absence of osteogenic induction, FGF-2–overexpressing MSCs retained an *FGFR2* expression profile comparable to that of Ct_ASCs, indicating that receptor downregulation reflects a reversible, signal-dependent adaptation rather than irreversible lineage commitment.

In the future, three-dimensional culture systems may further enhance the paracrine output of *FGF-2*–overexpressing, *TERT*-expressing MSCs, enabling the scalable production of CM and extracellular vesicles enriched with pro-angiogenic factors. Such cell-free products could be applied as off-the-shelf regenerative therapeutics that maximize angiogenesis while avoiding risks associated with cellular engraftment or unintended differentiation. This approach offers a practical and controllable strategy for vascularized tissue regeneration.

## Conclusion

In this study, we successfully established FGF-2-overexpressing adipose-derived stem cells (FGF-2_ASCs) and evaluated their dual roles in angiogenesis and osteogenesis. Overexpression of *FGF-2* significantly enhanced the secretion of pro-angiogenic factors and promoted endothelial cell migration and tube formation, primarily through the synergistic interaction between FGF-2 and VEGFA. However, *FGF-2* overexpression also markedly suppressed osteogenic differentiation, as evidenced by reduced matrix mineralization and downregulation of key osteogenic markers, including *ALP*, *OC*, and *FGFR2*. These findings indicate that FGF-2_ASCs function primarily as a potent source of pro-angiogenic paracrine factors rather than as direct contributors to bone formation. The CM derived from these cells exhibits robust angiogenic activity, supporting vascularization as a critical prerequisite for tissue regeneration. Accordingly, FGF-2_ASCs–derived CM represents a promising cell-free therapeutic strategy for vascularized tissue regeneration, offering enhanced efficacy, safety, and scalability for future regenerative medicine applications.

## Data Availability

The data presented in this study are available from the corresponding author on reasonable request.

## References

[1] Mastrullo, V., W. Cathery, E. Velliou, P. Madeddu, and P. Campagnolo. Angiogenesis in Tissue Engineering: As Nature Intended? *Front Bioeng Biotechnol.* 8:188, 2020. 10.3389/fbioe.2020.00188. 32266227 10.3389/fbioe.2020.00188 PMC7099606

[2] Masson-Meyers, D. S., and L. Tayebi. Vascularization strategies in tissue engineering approaches for soft tissue repair. *J Tissue Eng Regen Med.* 15(9):747–762, 2021. 10.1002/term.3225. 34058083 10.1002/term.3225 PMC8419139

[3] Song, H. H. G., R. T. Rumma, C. K. Ozaki, E. R. Edelman, and C. S. Chen. Vascular Tissue Engineering: Progress, Challenges, and Clinical Promise. *Cell Stem Cell.* 22(3):340–354, 2018. 10.1016/j.stem.2018.02.009. 29499152 10.1016/j.stem.2018.02.009 PMC5849079

[4] Ding, D.-C., W.-C. Shyu, and S.-Z. Lin. Mesenchymal Stem Cells. *Cell Transplantation.* 20(1):5–14, 2011. 10.3727/096368910X. 21396235 10.3727/096368910X

[5] Jin, S., C. Yang, J. Huang, L. Liu, Y. Zhang, S. Li, et al. Conditioned medium derived from FGF-2-modified GMSCs enhances migration and angiogenesis of human umbilical vein endothelial cells. *Stem Cell Res Ther.* 11(1):68, 2020. 10.1186/s13287-020-1584-3. 32070425 10.1186/s13287-020-1584-3 PMC7029497

[6] Matthew, A., and R. V. I. Nugent. Molecules in focus Fibroblast growth factor-2. *The International Journal of Biochemistry & Cell Biology.* 32:115–20, 2000. 10687947 10.1016/s1357-2725(99)00123-5

[7] Ferguson HR, Smith MP, Francavilla C. Fibroblast Growth Factor Receptors (FGFRs) and Noncanonical Partners in Cancer Signaling. *Cells*. 10(5)2021. 10.3390/cells10051201 10.3390/cells10051201 PMC8156822 34068954

[8] Ornitz, D. M., and N. Itoh. The Fibroblast Growth Factor signaling pathway. *Wiley Interdiscip Rev Dev Biol.* 4(3):215–266, 2015. 10.1002/wdev.176. 25772309 10.1002/wdev.176 PMC4393358

[9] Turner, N., and R. Grose. Fibroblast growth factor signalling: from development to cancer. *Nat Rev Cancer.* 10(2):116–129, 2010. 10.1038/nrc2780. 20094046 10.1038/nrc2780

[10] Prudovsky, I. Cellular Mechanisms of FGF-Stimulated Tissue Repair. *Cells.* 10(7):1830, 2021. 10.3390/cells10071830. 34360000 10.3390/cells10071830 PMC8304273

[11] Lotz, S., S. Goderie, N. Tokas, S. E. Hirsch, F. Ahmad, B. Corneo, et al. Sustained Levels of FGF2 Maintain Undifferentiated Stem Cell Cultures with Biweekly Feeding. *PLoS One.*8(2):e56289, 2013. 10.1371/journal.pone.0056289. 23437109 10.1371/journal.pone.0056289 PMC3577833

[12] Kitamura, M., M. Akamatsu, M. Machigashira, Y. Hara, R. Sakagami, T. Hirofuji, et al. FGF-2 stimulates periodontal regeneration: results of a multi-center randomized clinical trial. *J Dent Res.* 90(1):35–40, 2011. 10.1177/0022034510384616. 21059869 10.1177/0022034510384616

[13] Simons, M., B. H. Annex, R. J. Laham, N. Kleiman, T. Henry, H. Dauerman, et al. Pharmacological treatment of coronary artery disease with recombinant fibroblast growth factor-2: double-blind, randomized, controlled clinical trial. *Circulation.* 105(7):788–793, 2002. 10.1161/hc0802.104407. 11854116 10.1161/hc0802.104407

[14] Benington L, Rajan G, Locher C, Lim LY. Fibroblast Growth Factor 2-A Review of Stabilisation Approaches for Clinical Applications. *Pharmaceutics*. 12(6)2020. 10.3390/pharmaceutics12060508 10.3390/pharmaceutics12060508 PMC7356611 32498439

[15] Shen, C., P. Lie, T. Miao, M. Yu, Q. Lu, T. Feng, et al. Conditioned medium from umbilical cord mesenchymal stem cells induces migration and angiogenesis. *Mol Med Rep.* 12(1):20–30, 2015. 10.3892/mmr.2015.3409. 25739039 10.3892/mmr.2015.3409 PMC4438972

[16] Thummarati, P., and M. Kino-oka. Exogenous FGF-2 prolongs endothelial connection in multilayered human skeletal muscle cell sheet. *Journal of Bioscience and Bioengineering.* 131(6):686–95, 2021. 10.1016/j.jbiosc.2021.02.005. 33775542 10.1016/j.jbiosc.2021.02.005

[17] Li, Y., H. Zhu, C. Klausen, B. Peng, and P. C. K. Leung. Vascular Endothelial Growth Factor-A (VEGF-A) Mediates Activin A-Induced Human Trophoblast Endothelial-Like Tube Formation. *Endocrinology.* 156(11):4257–4268, 2015. 10.1210/en.2015-1228. 26327470 10.1210/en.2015-1228

[18] Pepper, M. S., N. Ferrara, L. Orci, and R. Montesano. Potent synergism between vascular endothelial growth factor and basic fibroblast growth factor in the induction of angiogenesis in vitro. *Biochem Biophys Res Commun.* 189(2):824–831, 1992. 10.1016/0006-291x(92)92277-5. 1281999 10.1016/0006-291x(92)92277-5

[19] Kano, M. R., Y. Morishita, C. Iwata, S. Iwasaka, T. Watabe, Y. Ouchi, et al. VEGF-A and FGF-2 synergistically promote neoangiogenesis through enhancement of endogenous PDGF-B–PDGFRβ signaling. *Journal of Cell Science.* 118(16):3759–3768, 2005. 10.1242/jcs.02483. 16105884 10.1242/jcs.02483

[20] Biel, N. M., and D. W. Siemann. Targeting the Angiopoietin-2/Tie-2 axis in conjunction with VEGF signal interference. *Cancer Lett.* 380(2):525–533, 2016. 10.1016/j.canlet.2014.09.035. 25312939 10.1016/j.canlet.2014.09.035 PMC4394020

[21] Wen, X., G. Hu, X. Xiao, X. Zhang, Q. Zhang, H. Guo, et al. FGF2 positively regulates osteoclastogenesis via activating the ERK-CREB pathway. *Archives of Biochemistry and Biophysics.* 727:109348, 2022. 10.1016/j.abb.2022.109348. 35835230 10.1016/j.abb.2022.109348

[22] Sakano, S., Y. Hasegawa, Y. Murata, T. Ito, E. Genda, H. Iwata, et al. Inhibitory effect of bFGF on endochondral heterotopic ossification. *Biochemical and Biophysical Research Communications.* 293(2):680–5, 2002. 10.1016/S0006-291X(02)00273-5. 12054522 10.1016/S0006-291X(02)00273-5

[23] Zhang, Y., L. Ling, D. O. A. A. Ajay, Y. M. Eio, A. J. van Wijnen, V. Nurcombe, et al. FGFR2 accommodates osteogenic cell fate determination in human mesenchymal stem cells. *Gene.*818:146199, 2022. 10.1016/j.gene.2022.146199. 35093449 10.1016/j.gene.2022.146199 PMC9256080

[24] Simann, M., S. Le Blanc, V. Schneider, V. Zehe, M. Lüdemann, N. Schütze, et al. Canonical FGFs Prevent Osteogenic Lineage Commitment and Differentiation of Human Bone Marrow Stromal Cells Via ERK1/2 Signaling. *J Cell Biochem.* 118(2):263–275, 2017. 10.1002/jcb.25631. 27305863 10.1002/jcb.25631

[25] Solchaga, L. A., K. Penick, J. D. Porter, V. M. Goldberg, A. I. Caplan, and J. F. Welter. FGF-2 enhances the mitotic and chondrogenic potentials of human adult bone marrow-derived mesenchymal stem cells. *J Cell Physiol.* 203(2):398–409, 2005. 10.1002/jcp.20238. 15521064 10.1002/jcp.20238

[26] Simonsen, J. L., C. Rosada, N. Serakinci, J. Justesen, K. Stenderup, S. I. Rattan, et al. Telomerase expression extends the proliferative life-span and maintains the osteogenic potential of human bone marrow stromal cells. *Nat Biotechnol.* 20(6):592–596, 2002. 10.1038/nbt0602-592. 12042863 10.1038/nbt0602-592

[27] Sahni, A., and C. W. Francis. Stimulation of endothelial cell proliferation by FGF-2 in the presence of fibrinogen requires αvβ3. *Blood.* 104(12):3635–3641, 2004. 10.1182/blood-2004-04-1358. 15297314 10.1182/blood-2004-04-1358

[28] Jia, X., and W. Liu. Vaccarin improves insulin sensitivity and glucose uptake in diet-induced obese mice via activation of GPR120-PI3K/AKT/GLUT4 pathway. *Biochemical and Biophysical Research Communications.* 634:189–95, 2022. 10.1016/j.bbrc.2022.09.099. 36252499 10.1016/j.bbrc.2022.09.099

[29] Murakami, M., L. T. Nguyen, Z. W. Zhang, K. L. Moodie, P. Carmeliet, R. V. Stan, et al. The FGF system has a key role in regulating vascular integrity. *The Journal of clinical investigation.* 118(10):3355–66, 2008. 18776942 10.1172/JCI35298 PMC2528913

[30] Han, Y., J. Yang, J. Fang, Y. Zhou, E. Candi, J. Wang, et al. The secretion profile of mesenchymal stem cells and potential applications in treating human diseases. *Signal Transduction and Targeted Therapy.* 7(1):92, 2022. 10.1038/s41392-022-00932-0. 35314676 10.1038/s41392-022-00932-0 PMC8935608

[31] Seghezzi, G., S. Patel, C. J. Ren, A. Gualandris, G. Pintucci, E. S. Robbins, et al. Fibroblast growth factor-2 (FGF-2) induces vascular endothelial growth factor (VEGF) expression in the endothelial cells of forming capillaries: an autocrine mechanism contributing to angiogenesis. *J Cell Biol.* 141(7):1659–1673, 1998. 10.1083/jcb.141.7.1659. 9647657 10.1083/jcb.141.7.1659 PMC2132998

[32] Hollborn, M., K. Jahn, G. A. Limb, L. Kohen, P. Wiedemann, and A. Bringmann. Characterization of the basic fibroblast growth factor-evoked proliferation of the human Müller cell line, MIO-M1. *Graefes Arch Clin Exp Ophthalmol.* 242(5):414–422, 2004. 10.1007/s00417-004-0879-x. 14963717 10.1007/s00417-004-0879-x

[33] Yanagita, M., Y. Kojima, M. Kubota, K. Mori, M. Yamashita, S. Yamada, et al. Cooperative effects of FGF-2 and VEGF-A in periodontal ligament cells. *J Dent Res.* 93(1):89–95, 2014. 10.1177/0022034513511640. 24186558 10.1177/0022034513511640 PMC3872850

[34] Murakami, M., L. T. Nguyen, K. Hatanaka, W. Schachterle, P. Y. Chen, Z. W. Zhuang, et al. FGF-dependent regulation of VEGF receptor 2 expression in mice. *J Clin Invest.* 121(7):2668–2678, 2011. 10.1172/jci44762. 21633168 10.1172/JCI44762 PMC3223828

[35] Boichuk S, Dunaev P, Galembikova A, Valeeva E. Fibroblast Growth Factor 2 (FGF2) Activates Vascular Endothelial Growth Factor (VEGF) Signaling in Gastrointestinal Stromal Tumors (GIST): An Autocrine Mechanism Contributing to Imatinib Mesylate (IM) Resistance. *Cancers (Basel)*. 16(17)2024. 10.3390/cancers16173103 10.3390/cancers16173103 PMC11394061 39272961

[36] Huang, H., A. Bhat, G. Woodnutt, and R. Lappe. Targeting the ANGPT–TIE2 pathway in malignancy. *Nature Reviews Cancer.* 10(8):575–585, 2010. 10.1038/nrc2894. 20651738 10.1038/nrc2894

[37] Hegen, A., S. Koidl, K. Weindel, D. Marmé, H. G. Augustin, and U. Fiedler. Expression of angiopoietin-2 in endothelial cells is controlled by positive and negative regulatory promoter elements. *Arterioscler Thromb Vasc Biol.* 24(10):1803–1809, 2004. 10.1161/01.ATV.0000140819.81839.0e. 15284088 10.1161/01.ATV.0000140819.81839.0e

[38] Mandriota, S. J., and M. S. Pepper. Regulation of angiopoietin-2 mRNA levels in bovine microvascular endothelial cells by cytokines and hypoxia. *Circ Res.* 83(8):852–859, 1998. 10.1161/01.res.83.8.852. 9776732 10.1161/01.res.83.8.852

[39] Kuroda, K., A. Sapadin, T. Shoji, R. Fleischmajer, and M. Lebwohl. Altered expression of angiopoietins and Tie2 endothelium receptor in psoriasis. *J Invest Dermatol.* 116(5):713–720, 2001. 10.1046/j.1523-1747.2001.01316.x. 11348459 10.1046/j.1523-1747.2001.01316.x

[40] Ikpegbu, E., L. Basta, D. N. Clements, R. Fleming, T. L. Vincent, D. J. Buttle, et al. FGF-2 promotes osteocyte differentiation through increased E11/podoplanin expression. *J Cell Physiol.* 233(7):5334–5347, 2018. 10.1002/jcp.26345. 29215722 10.1002/jcp.26345 PMC5900964

[41] Quarto, N., D. C. Wan, and M. T. Longaker. Molecular mechanisms of FGF-2 inhibitory activity in the osteogenic context of mouse adipose-derived stem cells (mASCs). *Bone.* 42(6):1040–1052, 2008. 10.1016/j.bone.2008.01.026. 18420480 10.1016/j.bone.2008.01.026

[42] Gromolak S, Krawczenko A, Antonczyk A, Buczak K, Kielbowicz Z, Klimczak A. Biological Characteristics and Osteogenic Differentiation of Ovine Bone Marrow Derived Mesenchymal Stem Cells Stimulated with FGF-2 and BMP-2. *Int J Mol Sci*. 21(24)2020. 10.3390/ijms21249726 10.3390/ijms21249726 PMC7766718 33419255

[43] Xue, G., H. L. Yan, Y. Zhang, L. Q. Hao, X. T. Zhu, Q. Mei, et al. c-Myc-mediated repression of miR-15-16 in hypoxia is induced by increased HIF-2α and promotes tumor angiogenesis and metastasis by upregulating FGF2. *Oncogene.* 34(11):1393–1406, 2015. 10.1038/onc.2014.82. 24704828 10.1038/onc.2014.82

[44] Ornitz, D. M., and P. J. Marie. Fibroblast growth factor signaling in skeletal development and disease. *Genes Dev.* 29(14):1463–1486, 2015. 10.1101/gad.266551.115. 26220993 10.1101/gad.266551.115 PMC4526732

