# Publisher Correction: FGF-2–Overexpressing Adipose-Derived Stem Cells as a Paracrine Platform for Angiogenesis-Driven Tissue Regeneration

**DOI:** 10.1007/s12195-026-00902-4

**Published:** 2026-04-16

**Authors:** Daisuke Seki, Michiyo Honda

**Affiliations:** Department of Applied Chemistry, School of Science and Technology, Meiji University, 1-1-1 Higashimita, Tama-ku, Kawasaki, Kanagawa 214-8571 Japan

**Publisher Correction to: Cellular and Molecular Bioengineering (2025) 19:73–87** 10.1007/s12195-025-00883-w

The editorial responsibility footnote is incorrect. It should state that Associate Editor Alice Anna Tomei oversaw the review of this article.

